# Prognostic significance of peritumoral vascular invasion in breast cancer.

**DOI:** 10.1038/bjc.1984.255

**Published:** 1984-12

**Authors:** R. Bettelheim, H. G. Penman, H. Thornton-Jones, A. M. Neville

## Abstract

A prospective study of 232 patients with primary invasive breast cancer (UICC Stages I, II and III) and histologically confirmed axillary node status was carried out to assess the prognostic significance of several readily available clinical and pathological characteristics. In addition to the recognised utility of tumour size and axillary lymph node status, the presence or absence of cohesive clumps of malignant cells in peritumoral vascular spaces (both lymphatic and blood vessels) was found to be prognostically important.


*Br. J. Cancer* (1984), **50**, 771–777

### Prognostic significance of peritumoral vascular invasion in breast cancer

R. Bettelheim[1], H.G. Penman[2], H. Thornton-Jones[3] & A.M. Neville[4]

[1]*South West Thames Regional Cancer Organization, Royal Marsden Hospital (Surrey Branch), Downs Road, Sutton, Surrey;* [2]*Department of Pathology, Crawley Hospital, Crawley, Sussex;* [3]*The South Thames Cancer Registry, Sutton, Surrey; and* [4]*Ludwig Institute for Cancer Research, London Branch, Haddow Laboratories, Sutton, Surrey, UK.*

**Summary** A prospective study of 232 patients with primary invasive breast cancer (UICC Stages I, II and III) and histologically confirmed axillary node status was carried out to assess the prognostic significance of several readily available clinical and pathological characteristics. In addition to the recognised utility of tumour size and axillary lymph node status, the presence or absence of cohesive clumps of malignant cells in peritumoral vascular spaces (both lymphatic and blood vessels) was found to be prognostically important.

The overall survival rate in breast cancer has not improved over the last few decades, despite better radiotherapy techniques and a wide range of new hormonal and cytotoxic agents (Brinkley *et al.*, 1984; Powles *et al.*, 1980). It is accepted that a proportion of cases with so called "localised" mammary carcinomas have systemic disease at the time of initial presentation which currently available physical and biochemical methods fail to detect. It is, therefore, important to have reliable ways of identifying those patients most at risk of developing metastatic disease who might consequently benefit by receiving systemic adjuvant therapy following primary surgical care. In the absence of a single reliable prognostic criterion, varying combinations of parameters may be used (Fisher *et al.*, 1975; Report of the Primary Therapy of Breast Cancer Study Groups, 1978; Hutter, 1980; Rosen *et al.*, 1982). These include the size of the primary tumour, its histological features and oestrogen receptor content, the degree of elastosis and small round cell infiltration, and most important of all, axillary lymph node involvement determined pathologically. However, these are far from adequate so that there is still a need for other prognostic indices.

Using recurrence rate (disease-free survival) as the end point, we have found that the presence of tumour emboli in vascular spaces associated with primary breast cancer carries a bad prognosis comparable in status to axillary lymph node involvement.

### Patients

A total of 232 consecutive patients with UICC Stage I, II or III invasive breast carcinomas were entered into the study carried out at the Royal Marsden Hospital (Surrey Branch), St Helier, Crawley and Redhill General Hospitals between September 1976 and March 1980. The mean follow-up period is 64.5 months, (range, 45–84 months). The mean age of the patients is 57.9 years (range, 27–88 years). Seventy-one percent of the patients were post-menopausal (at least 1 year after their last menstrual period), 21% were menstruating regularly and 8% were showing signs of "incipient'

**Table I.** Patient and pathological data of the 232 patients in the series.

| | |
|---|---|
| No. of patients | 232 |
| Age (years) | |
| mean | 57.9 |
| range | 27–88 |
| Menopausal status | |
| pre- | 50 |
| peri- | 17 |
| post- | 165 |
| Tumour site | |
| left | 119 |
| right | 109 |
| bilateral | 4 |
| Tumour size (cm) | |
| mean | 2.7 |
| range | 0.4–12.0 |
| 3 and <3 | 176 |
| >3 | 56 |
| Surgery | |
| local excision with axillary biopsy | 29 |
| mastectomy with axillary clearance | 203 |
| Histology (all inflitrating carcinomas) | |
| ductal | 223 |
| lobular | 4 |
| papillary | 1 |
| mucinous | 3 |
| medullary | 1 |

Correspondence: R. Bettelheim
Received 8 June 1984; accepted 14 August 1984

© The Macmillan Press Ltd., 1984

menopause at the time of presentation (peri-menopausal) (Table I).

All patients had a blood sample taken and the ESR, carcinoembryonic antigen, alkaline phosphatase and gamma-glutamyl transpeptidase levels were measured. Only patients admitted to the Royal Marsden Hospital and St. Helier Hospital had a liver ultrasound, Technetium-99 bone scan and skeletal survey as part of the pre-operative investigations.

All patients had their primary tumours removed surgically and the ipsilateral axillae explored. The extent of surgical treatment ranged from local excision with axillary biopsy to modified radical mastectomy. The majority of patients were treated by simple mastectomy with lower axillary clearance (Table I). All patients with medial and/or central primary tumours (regardless of axillary status) and patients with pathologically confirmed axillary nodal involvement, received post-operative radiotherapy. The progress of all patients was monitored on an out-patient basis: quarterly in the first post-operative year, biannually for the next 2 years and thereafter annually. Both local recurrences and the detection of distant metastases were considered treatment failures (Karabali-Dalamaga *et al.*, 1978; Meir *et al.*, 1980).

### Methods

All the material was examined immediately after operation. The tumour(s) were measured and samples with a generous portion of surrounding breast tissue taken. In addition, the nipple and mammary tissue from each quadrant distant from the main mass were examined. The axillae were dissected and each lymph node taken for histology.

All the tissues were fixed in neutral buffered formalin and processed for light microscopy and staining with H and E by conventional methods. Between one and three sections from each tumour and one section of each lymph node were examined. Steroid receptor analyses were not available at the outset of this study.

In some instances, immunocytochemical methods were used to outline vascular and lymphatic channels (Bettelheim *et al.*, 1983). The Logrank test as described by Peto *et al.* (1977) was used for all statistical analyses.

### Histology

The pathological types of the 232 primary tumours examined are shown in Table I. Of the ductal carcinomas, 140 were of grade 2 and 83 of grade 3 (Bloom & Richardson, 1957). None was considered to be of Grade 1. Interestingly, in this study, lobular carcinomas were less frequent than would have been expected (Fisher *et al.*, 1975). Other pathological features noted were the presence or absence of tumour cells in vascular structures in proximity to the dominant mass, desmoplasia, small round cell infiltration, the presence or absence of micro-calcification, the presence or absence of elastosis, the number of lymph nodes involved with tumour and the extent of such involvement: minimal (single clump of malignant cells); $<1/3$ of the lymph node mass as seen on the histological section; $>1/3$ of the lymph node; extention through the nodal capsule. In cases with multiple nodal metastases showing various extent of involvement only the most extensive was charted.

### Results

The patient data with potential prognostic significance were submitted to multiparametric analysis. The parameters were assessed singly and in combination for disease-free survival. Clinical factors such as age, menopausal status, tumour side, or site were found not to be significant, as was the type of surgical intervention, i.e. local excision with axillary sampling as opposed to mastectomy with axillary exploration (Table II). Overall of 29 patients subjected to local excision and axillary sampling, 14 (48%) developed recurrences, while of the 203 who had a mastectomy 82 (40%) developed recurrences. However, 11/29 (38%) patients with axillary sampling had involved nodes; 112/203 (55%) patients who had a mastectomy with axillary clearance were found to have nodal involvement. Tumour-associated substances (ESR, carcino-embryonic antigen, alkaline phosphatase and gamma-glutamyl transpeptidase) measured pre-operatively did not help to identify the patients likely to develop recurrent disease (Lawrence & Neville 1983). Pathological data such as histological tumour grade (Bloom & Richardson, 1957), desmoplasia, elastosis, small round cell infiltration and the presence or absence of tumour microcalcification were not reflected in the disease free survival. Three pathological factors, however, were shown to be of prognostic value: tumour size, axillary lymph node involvement and the presence of cohesive clumps of tumour cells in peritumoral vascular spaces (Bettleheim *et al.*, 1983; Bettelheim & Neville, 1981). The statistical results are shown in Figures 1, 2 and 3 and Table II.

The disease-free survival in patients presenting with tumours measuring $<3$ cm was significantly better compared to those whose tumours were $>3$ cm in their longest axis (chi-square $=4.13$; $P<0.05$).

**Table II.** Incidence of lymph node metastases as a function of operative procedure and resulting outcome.

| Prognostic factors | Local excision[a] No. of patients | Local excision[a] No. of recurrences (%) | Mastectomy[a] No. of patients | Mastectomy[a] No. of recurrences (%) |
|---|---|---|---|---|
| Lymph node −ve | 18 | (38.8) | 91 | (24) |
| (LN) +ve | 11 | (63.6) | 112 | (53.5) |
| Vascular invasion −ve | 14 | (42.8) | 102 | (27.4) |
| (VI) +ve | 15 | (53.3) | 101 | (53.4) |
| LN− VI− | 9 | (33.3) | 65 | (18.4) |
| LN− VI+ | 9 | (44) | 26 | (38.4) |
| LN+ VI− | 5 | (60) | 37 | (43) |
| LN+ VI+ | 6 | (66) | 75 | (58.6) |

[a]11/29 (38%) of patients with axillary sampling had lymph node involvement while 112/203 (55%) with axillary clearance had involved nodes.

**Figure 1** Disease free survival by tumour size: ------ tumours measuring 3 cm and <3 cm (176 cases); ——— tumours measuring >3 cm (56 cases). The difference in disease free survival is significant (chi square = 4.13; $P<0.05$).

**Figure 2** Disease free survival by axillary lymph node status: ———— axillary lymph nodes free of tumour (117 cases); ------ axillary lymph nodes involved with tumour (115 cases). The difference in disease free survival is significant (chi square=21,74; $P<0.005$).

**Figure 3** Disease free survival by vascular invasion: ------ no tumour cells identified in peritumoral lymphatic and blood vessels (117 cases); ———— tumour emboli seen in peritumoral vascular spaces (115 cases). The difference in disease free survival is significant (chi-square 20.98; $P<0.005$).

Axillary lymph node involvement in breast cancer irrespective of degree (Table II), was associated with a recurrence rate of 54.4% in contrast to 26.8% in cases with negative axillary lymph nodes. The difference in disease-free survival between axillary lymph node positive and axillary lymph node negative patients was statistically significant (chi-square = 20.26, $P<0.005$). There were only 7 cases of local recurrence without distant metastases in this study and all occurred in patients whose axillary nodes were uninvolved. No relation to the type of surgical procedure was apparent: Five patients were treated by a mastectomy, 2 had their tumours removed locally.

The invasion by tumour cells of the vascular structures in proximity to the primary lesion was of similar significance with a recurrence rate of 53.4% when used as a separate criterion. The recurrence rate in tumours where vascular invasion was not detected was 29.5%. The difference in disease-free survival between these two groups was found to be significant (chi-square = 20.98; $P<0.005$). When vascular invasion was adjusted for both tumour size and axillary status, it retained its prognostic significance (chi-square 9.2, $P<0.01$).

By combining axillary nodal status with vascular invasion we were able to show that peritumoral vascular invasion identified in patients with axillary lymph nodes free of tumour is a significant prognostic factor (chi-square 9.85, $P<0.005$). In patients with axillary lymph nodes involved with tumour, however, the situation is less clear-cut: the contrast between cases with peritumoral vascular invasion present and cases where peritumoral vascular invasion was absent, approached but did not quite achieve significance at the 5% level with chi-square = 3.68 (Figures 4 and 5).

### Discussion

There is a need to refine and identify further factors which assist in the prognostic assessment of patients with breast cancer. Moreover, the identification of indices which are available to all surgeons and pathologists would be of particular value as not everyone has access to steroid receptor analyses, esoteric screening methods, studies of tumour ploidy, etc. Our study has confirmed the validity of tumour size and axillary nodal status while drawing attention to the further prognostic importance of the presence of tumour cells in endothelial-lined spaces – both lymphatic channels

**Figure 4** Disease free survival in axillary lymph node negative patients by vascular invasion: ------ no tumour cells identified in peritumoral vascular spaces (76 cases); ———— tumour emboli seen in vascular spaces (41 cases). The difference in disease free survival is significant (chi-square 9.85; $P<0.005$).

**Figure 5** Disease free survival in axillary lymph node positive patients by vascular invasion: ------ no tumour cells present in peritumoral vascular spaces (41 cases); ———— tumour emboli present in vascular spaces. The difference in disease free survival is not significant (chi-square 3.68; $P<0.01$).

and small blood vessels – closely associated with primary invasive mammary carcinoma. This matter has so far received only scant attention (Bettelheim *et al.*, 1983; Bettelheim & Neville, 1981; Kister *et al.*, 1966; Nime *et al.*, 1977; Roberts & Hahnel, 1981; Rosen *et al.*, 1981, 1982). Routine H & E histological preparations are quite adequate for the detection of vascular invasion by malignant cells provided a generous stromal edge to the tumour is taken (Bettelheim, *et al.*, 1983). Our previous study employed immunocytochemistry and various antibodies to outline vascular and lymphatic channels. This approach, although aesthetically pleasing, did not increase the detection rate of such intravascular tumour emboli. It did, however, identify some small blood vessels interpreted as lymphatic channels on routine preparations stained by H & E (Report of the Primary Therapy of Breast Cancer Study Groups, 1978).

In this study with a median follow-up of around 5 years, the invasion by tumour cells of lymphatic and blood vessels associated with the primary tumour, provided prognostic information equal to that derived from axillary lymph node involvement. A recurrence rate of 40% in the group of patients with no axillary involvement but with intratumoral vascular invasion contrasts with the 20.5% recurrence rate from patients in whom neither nodal nor vascular invasion was observed. The assessment of vascular invasion, therefore, provides important additional information in cases where axillary lymph nodes are available for histological examination. It is likely to become even more valuable as a further prognostic parameter as more and more surgeons practice local excisions without axillary exploration for breast cancer.

In another separate, but related study, we have found the presence of tumour cells in the bone marrow in about 26% of patients at the time of their initial presentation with breast cancer and when all other diagnostic procedures failed to reveal evidence of metastases (Sloane *et al.*, 1980). Preliminary analyses have shown a relationship between the presence of such marrow micrometastases and peritumoral vascular and/or lymphatic invasion (Dearnaley *et al.*, 1981; Redding *et al.*, 1983). Accordingly the finding of such invasion may present an important feature for the stratification of patients entering therapy trials and may indicate the need for active adjuvant intervention.

We are deeply grateful to the following for their support and generous help:

Messrs J.C. Gazet, Royal Marsden Hospital, Surrey Branch; A. Nash, St. Helier Hospital; J. Neely and J. Bull, Crawley Hospital; I.D. Hunter-Craig and J. Hale, Redhill General Hospital; Drs. T. J. Powles, R.C. Coombes and J.P. Sloane, Royal Marsden Hospital, Surrey Branch; R.L. Carter, Institute of Cancer Research; T. Goodier and J. Bensted, St. Helier Hospital; T.A.J. Wickham, Crawley Hospital and Redhill General Hospital; the technical staff of the Departments of Histopathology, Haematology and Medical Records in the Royal Marsden Hospital, Surrey Branch, St. Helier, Crawley Hospital and Redhill General Hospital.

We thank the South West Thames Regional Cancer Organization and Drs. G. Taylor and J. Chamberlain for the initiation and partial financial support for the study.

We thank Mrs. H. Spreadborough for typing the manuscript.

### References

BETTELHEIM, R. MITCHELL, D & GUSTERSON, B.A (1983). The role of immunocytochemistry to identify vascular invasion in breast cancer. *J. Clin. Pathol.*, **37**, 364.

BETTELHEIM, R. & NEVILLE, A.M. (1981). Lymphatic and vascular channel involvement within infiltrative breast carcinomas as a guide to prognosis at the time of primary surgical treatment. *Lancet*, **ii**, 631.

BLOOM, H.J.G. & RICHARDSON, W.W. (1957). Histological grading and prognosis in breast cancer. *Br. J. Cancer*, **11**, 359.

BRINKLEY, D., HARBITTLE, J.L. & HOUGHTON, J. (1984). The Cancer Research Campaign (King's/Cambridge) trial for early breast cancer: An analysis of the radiotherapy data. *Br. J. Radiol.*, **57**, 307.

DEARNALEY, D.P., ORMEROD, M.G., SLOANE, J.P. & 8 others (1981). Increased detection of mammary carcinoma cells in marrow smears using antisera to epithelial membrane antigen. *Br. J. Cancer*, **44**, 85.

FISHER, E.R., GREGORIO, R.M. & FISHER, B. (1975). The pathology of invasive breast cancer. *Cancer*, **36**, 1.

HUTTER, R.V.P. (1980). The influence of pathologic factors on breast cancer management. *Cancer*, **46**, 961.

KISTER, S.J., SOMMERS, S.C., HAAGENSEN, C.D. & COOLEY, E. (1966). Revaluation of blood vessel invasion as a prognostic factor in carcinoma of the breast. *Cancer*, **19**, 1213.

LAURENCE, D.J.R. & NEVILLE, A.M. (1983). The detection and evaluation of human tumour metastases. *Cancer Metastasis Rev.*, **2**, 351.

NIME, F., ROSEN, P.P., THALER, H.T., ASHIKARI, R. & URBAN, J.A. (1977). Prognostic significance of tumor emboli in intramammary lymphatics in patients with mammary carcinoma. *Am. J. Surg. Pathol.* **1**, 25.

PETO, R., PIKE, M.C., BRESLOW, N.E. & 6 others (1977). Design and analysis of randomized clinical trials requiring prolonged observation of each patient. II. Analysis and examples. *Br. J. Cancer*, **35**, 1.

POWLES, T.J., SMITH, I.E., FORD, H.T. COOMBES, R.C. JONES, J.M. & GAZET, J.-C. (1980). Failure of chemotherapy to prolong survival in a group of patients with metastatic breast cancer. *Lancet*, **i**, 580.

REDDING, W.H., COOMBES, R.C., MONAGHAN, P. & 7 others (1983). Detection of micrometastases in patients with primary breast cancer. *Lancet*, **ii**, 1271.

Report of the Primary Therapy of Breast Cancer Study Groups. (1978). Identification of breast cancer patients with high risk of early recurrence after radical mastectomy. II. Clinical and pathological correlations. *Cancer*, **42**, 2809.

ROBERTS, A.N. & HAHNEL, R. (1981). Oestrogen receptor assay and morphology of breast cancer. *Pathology*, **13**, 317.

ROSEN, P.P., SAIGO, P.E., BRAUN, D.W., Jr., BEATTIE, E.J. & KINNE, D.W. (1982). Occult axillary lymph node metastases from breast cancers with intramammary lymphatic tumor emboli. *Am. J. Surg. Pathol.* **6**, 639.

ROSEN, P.P., SAIGO, P.E., BRAUN, D.W., Jr., WEATHERS, E. & DE PALO, A. (1981). Predictors of recurrence in stage 1 (T1NoMo) breast carcinoma. *Annals of Surg.*, **193**, 15.

SLOANE, J.P., ORMEROD, M.G., IMRIE, S.F. & COOMBES, R.C. (1980). The use of antisera to epithelial membrane antigen in detecting micrometastases in histological sections. *Br. J. Cancer*, **42**, 392.


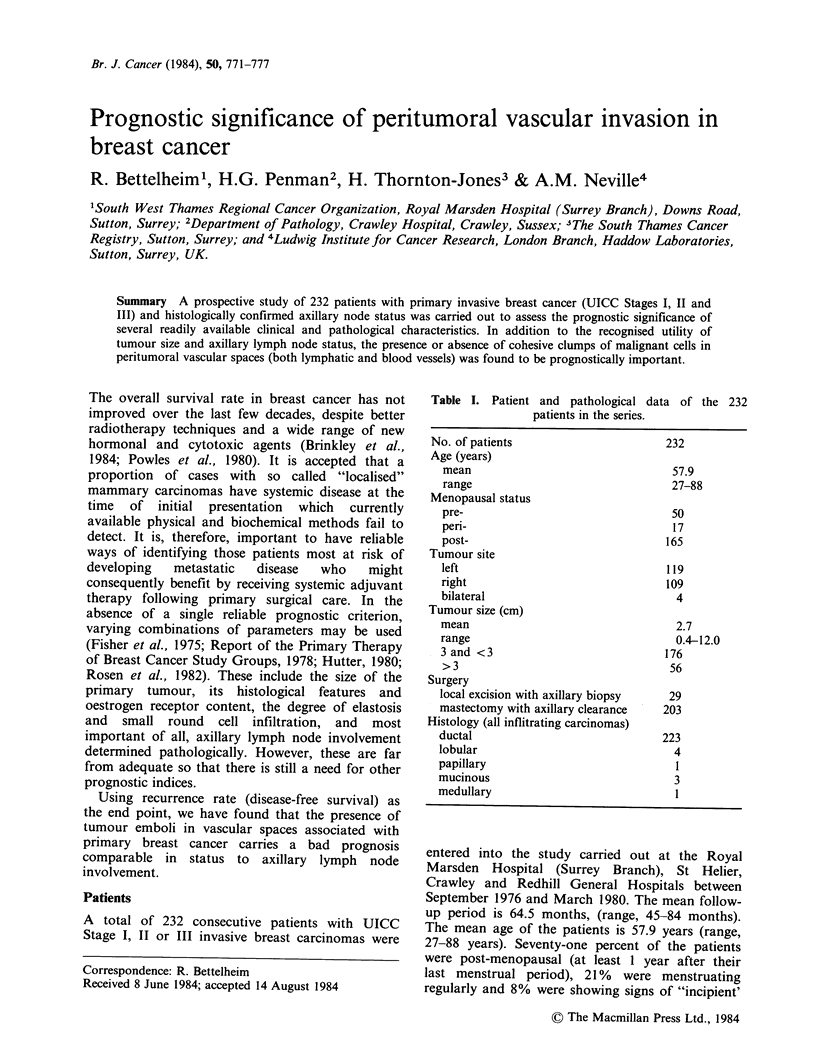

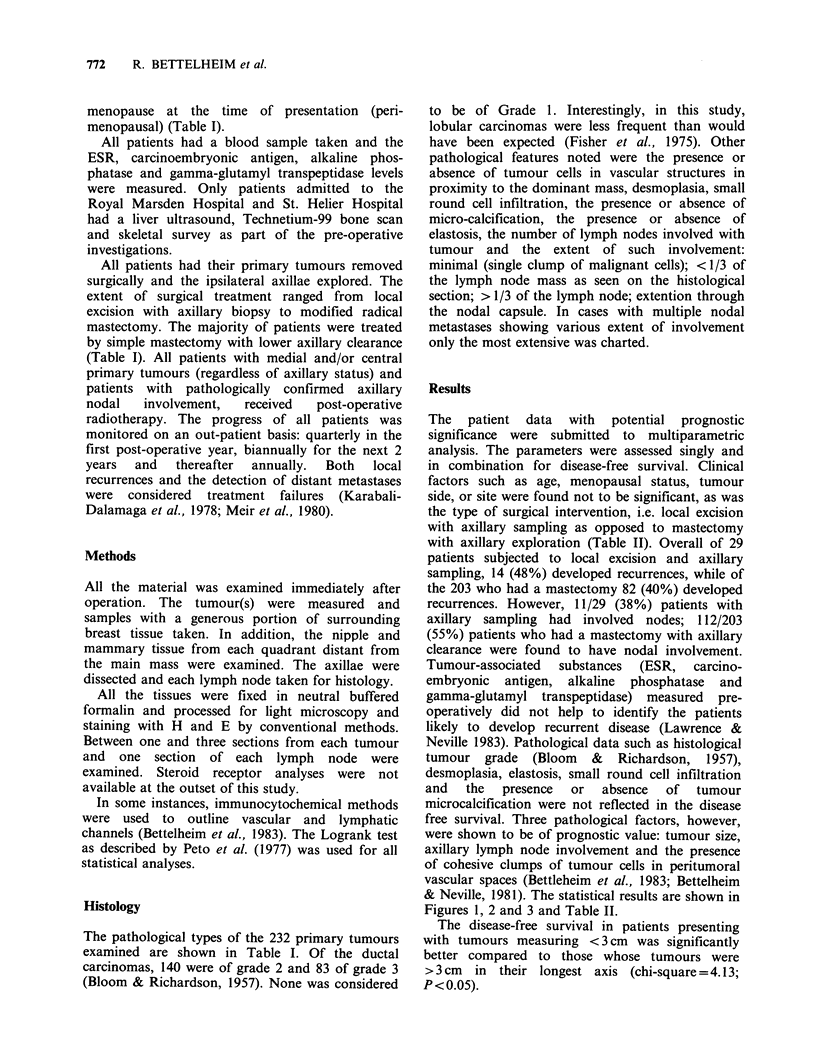

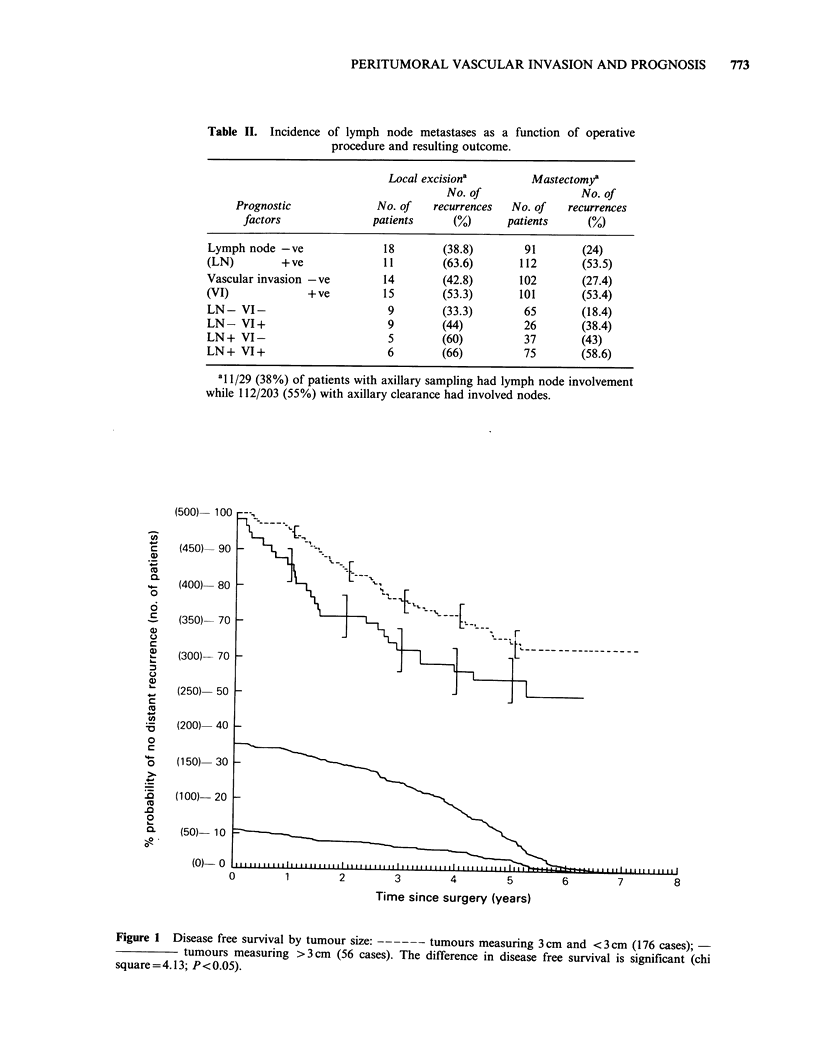

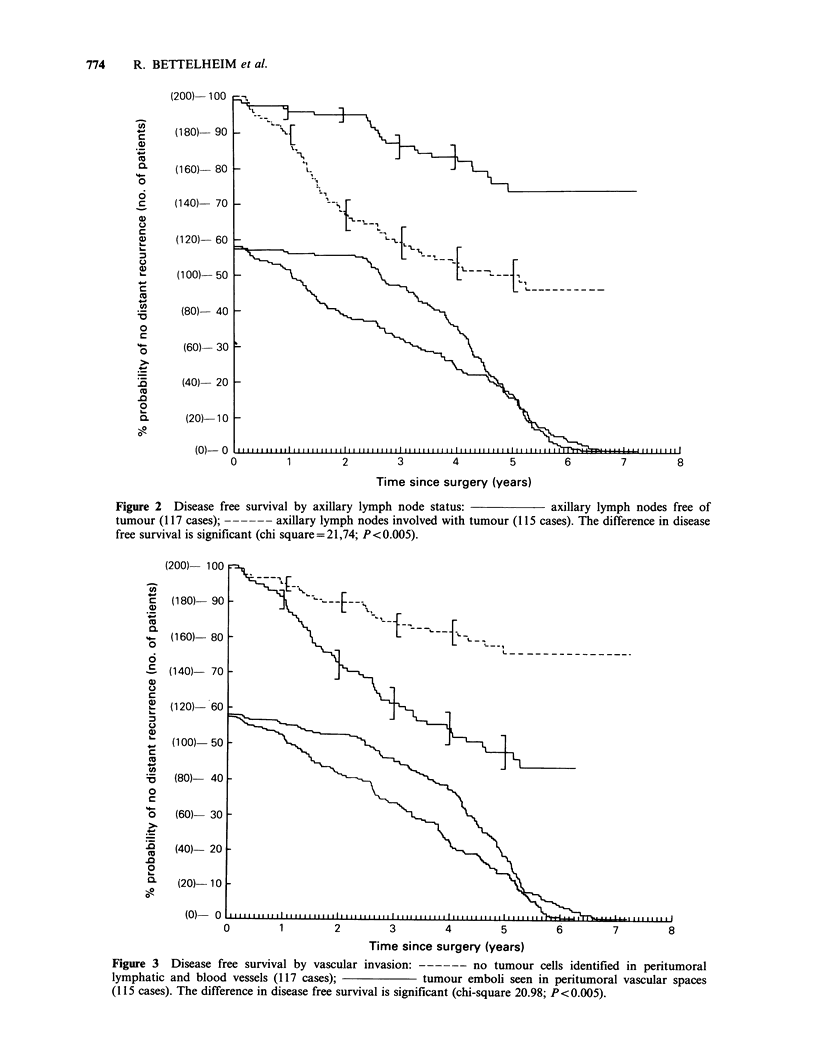

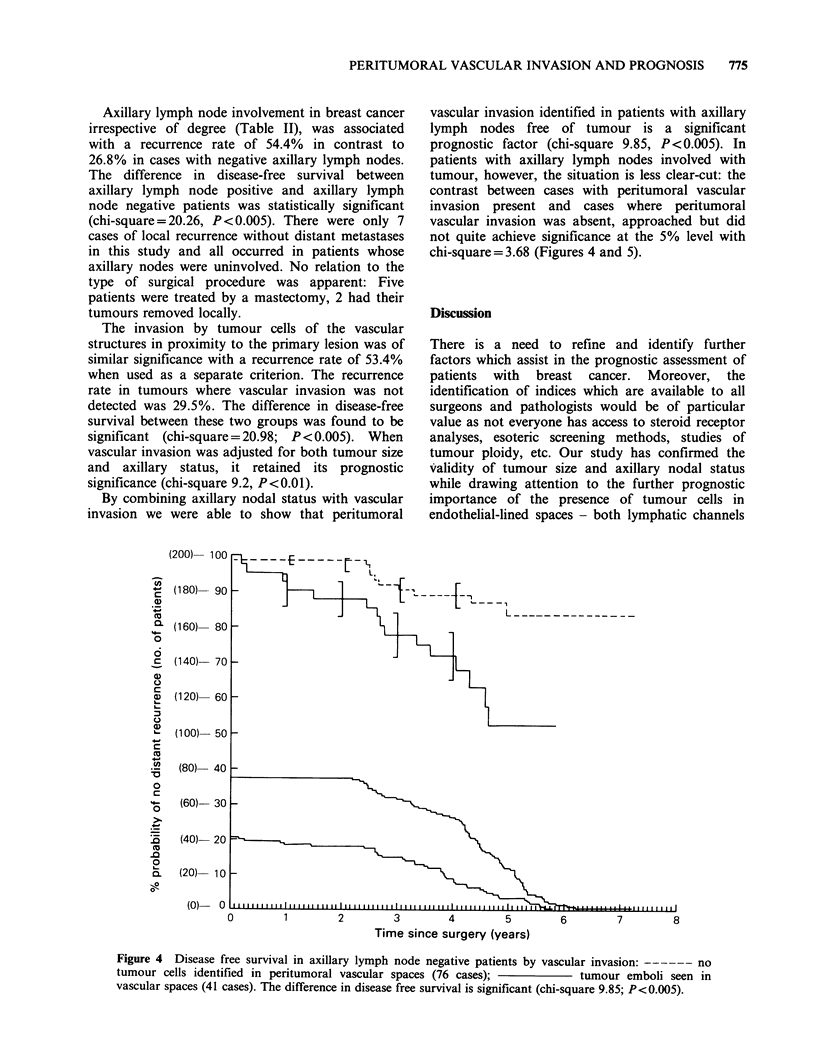

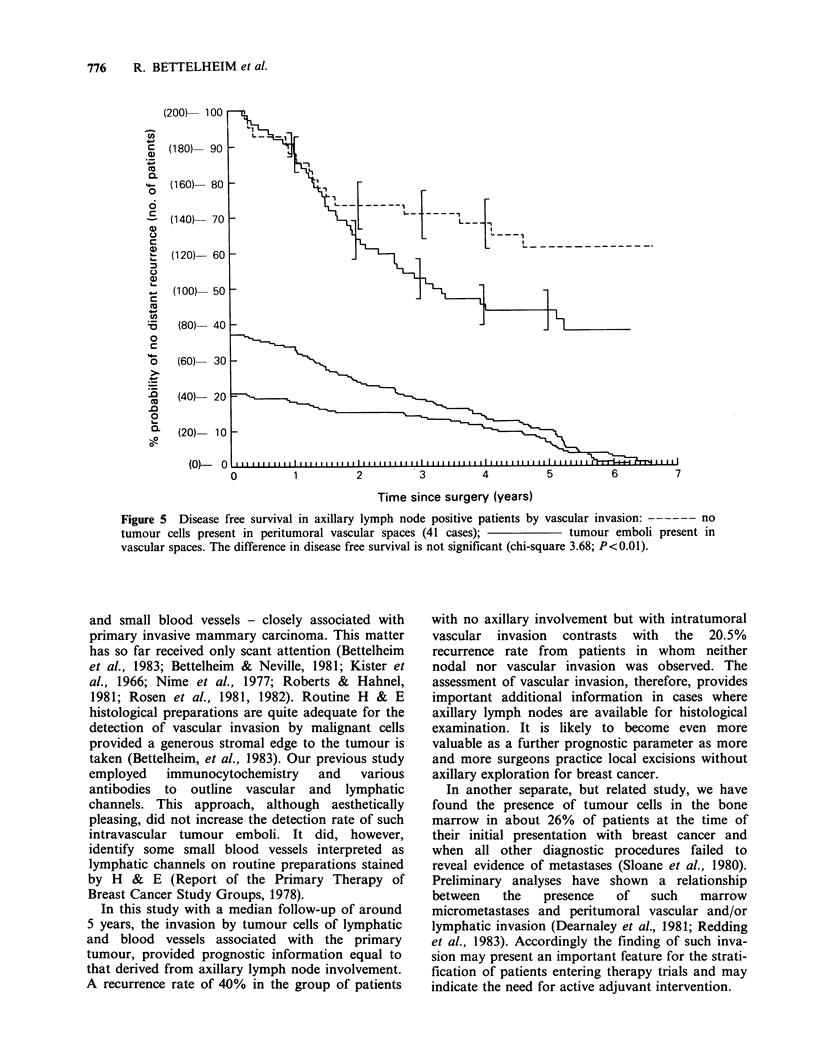

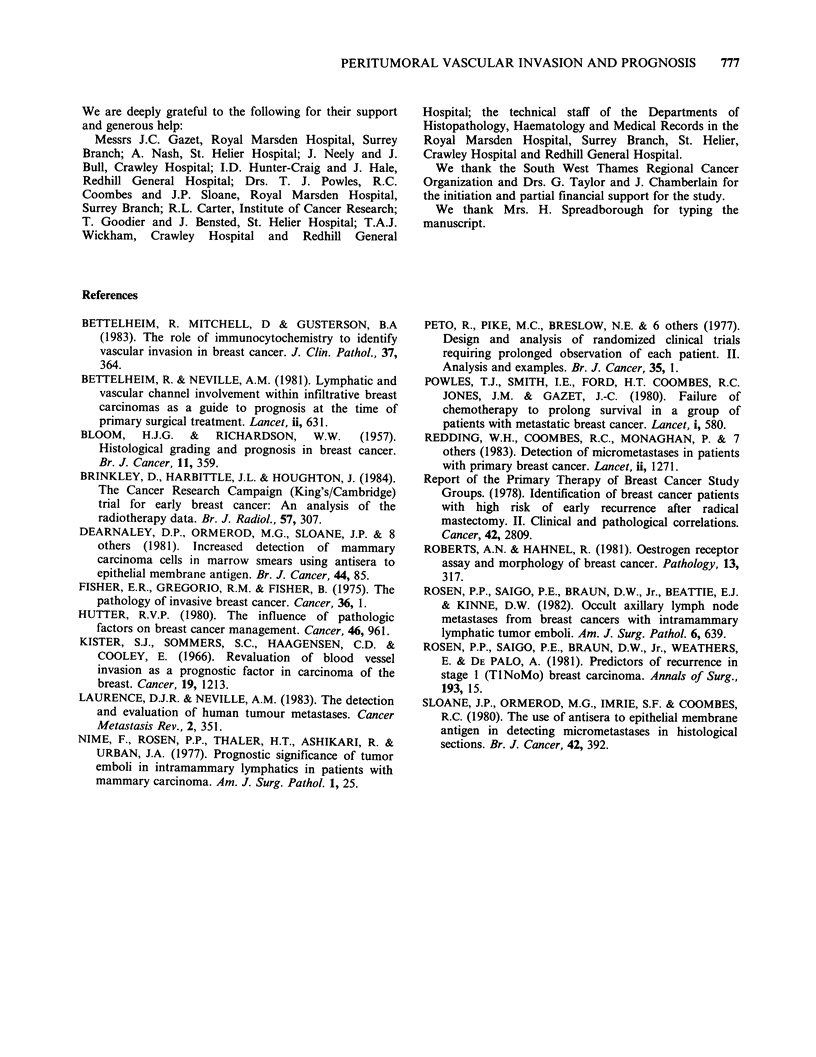


## References

[OCR_00715] BLOOM H. J., RICHARDSON W. W. (1957). Histological grading and prognosis in breast cancer; a study of 1409 cases of which 359 have been followed for 15 years.. Br J Cancer.

[OCR_00709] Bettelheim R., Neville A. M. (1981). Lymphatic and vascular channel involvement within infiltrative breast carcinomas as a guide to prognosis at the time of primary surgical treatment.. Lancet.

[OCR_00732] Fisher E. R., Gregorio R. M., Fisher B., Redmond C., Vellios F., Sommers S. C. (1975). The pathology of invasive breast cancer. A syllabus derived from findings of the National Surgical Adjuvant Breast Project (protocol no. 4).. Cancer.

[OCR_00736] Hutter R. V. (1980). The influence of pathologic factors on breast cancer management.. Cancer.

[OCR_00740] Kister S. J., Sommers S. C., Haagensen C. D., Cooley E. (1966). Re-evaluation of blood-vessel invasion as a prognostic factor in carcinoma of the breast.. Cancer.

[OCR_00746] Laurence D. J., Neville A. M. (1983). The detection and evaluation of human tumor metastases.. Cancer Metastasis Rev.

[OCR_00751] Nime F. A., Rosen P. P., Thaler H. T., Ashikari R., Urban J. A. (1977). Prognostic significance of tumor emboli in intramammary lymphatics in patients with mammary carcinoma.. Am J Surg Pathol.

[OCR_00765] Powles T. J., Coombes R. C., Smith I. E., Jones J. M., Ford H. T., Gazet J. C. (1980). Failure of chemotherapy to prolong survival in a group of patients with metastatic breast cancer.. Lancet.

[OCR_00769] Redding W. H., Coombes R. C., Monaghan P., Clink H. M., Imrie S. F., Dearnaley D. P., Ormerod M. G., Sloane J. P., Gazet J. C., Powles T. J. (1983). Detection of micrometastases in patients with primary breast cancer.. Lancet.

[OCR_00781] Roberts A. N., Hähnel R. (1981). Oestrogen receptor assay and morphology of breast cancer.. Pathology.

[OCR_00786] Rosen P. P., Saigo P. E., Braun D. W., Beattie E. J., Kinne D. W. (1982). Occult axillary lymph node metastases from breast cancers with intramammary lymphatic tumor emboli.. Am J Surg Pathol.

[OCR_00792] Rosen P. P., Saigo P. E., Braun D. W., Weathers E., DePalo A. (1981). Predictors of recurrence in stage I (T1N0M0) breast carcinoma.. Ann Surg.

[OCR_00798] Sloane J. P., Ormerod M. G., Imrie S. F., Coombes R. C. (1980). The use of antisera to epithelial membrane antigen in detecting micrometastases in histological sections.. Br J Cancer.

